# Supplementary material for: Grafting of iPS cell-derived tenocytes promotes motor function recovery after Achilles tendon rupture
Source: Nat Commun. 2021 Aug 18;12:5012. doi: 10.1038/s41467-021-25328-6 (PMC8373964; doi:10.1038/s41467-021-25328-6)
Supplement: Supplementary file 1 — Supplementary information [file 41467_2021_25328_MOESM1_ESM.pdf]

Supplementary Information

Supplementary Figure 1. Proliferative properties of iPSC-syndetome

Supplementary Figure 2. Single-cell RNA sequencing throughout iPSC-syndetome differentiation

Supplementary Figure 3. Load-displacement responses of repaired tendon

Supplementary Figure 4. Therapeutic effects of iPSC-derived sclerotome, BMSC, and recombinant TGFβ3, IGF1 proteins

Supplementary Table 1. Primer sequences for qPCR analysis

Supplementary Table 2. Antibodies for immunostaining

a

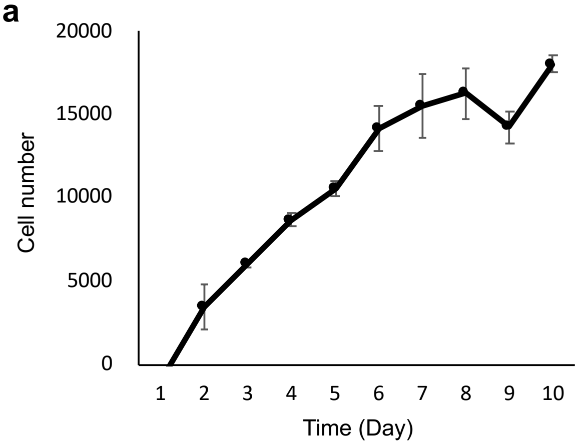

Figure S1. Proliferative properties of iPSC-syndetome

a, Cell growth curve of iPSC-syndetome. Differentiated iPSC-syndetome were distributed at a count of 2x10^3 cells per well into 96 well-plates then counted the number of cells every 24 hrs using Cell Counting Kit-8 (Dojindo Molecular Technologies) according to the manufacturer's protocol. Data represent mean ± SE (n = 3: biologically independent samples). Source data are provided as a Source Data file.

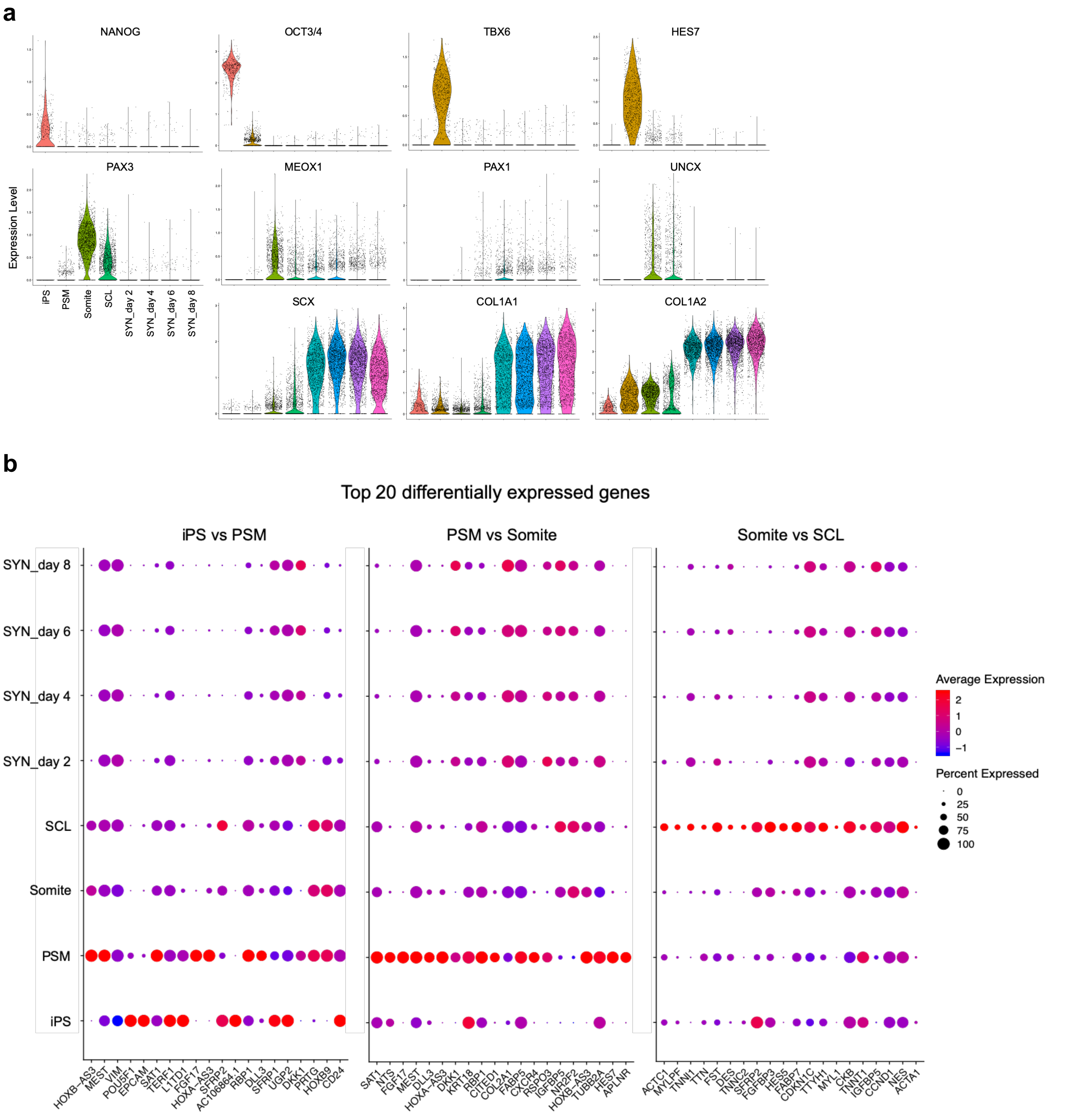

Figure S2. Single-cell RNA sequencing throughout iPSC-syndetome differentiation

a, Violin plot analysis of iPSCs, presomitic mesoderm, somite, sclerotome, and syndetome. Gene markers for iPSCs (*NANOG, OCT3/4*), presomite (*TBX6*, *HES7*), somite (*PAX3*, *MEOX1*), sclerotome (*PAX1*, *UNCX*), syndetome (*SCX*, *COL1A1*, *COL1A2*) were used. **b,** Dot plot analysis showing top 20 genes that differentially expressed in iPSCs and presomitic mesoderm; presomitic mesoderm and somite; somite and sclerotome. iPSC, induced pluripotent stem cell; PSM, presomitic mesoderm; SCL, sclerotome; SYN, syndetome.

a

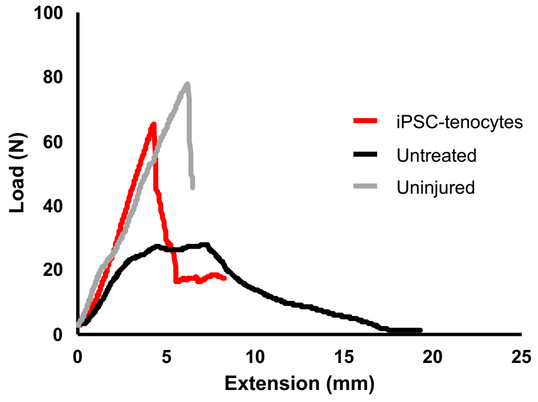

Figure S3. Load-displacement responses of repaired tendon

a, Representative load-displacement curves of repaired tendon in iPSC-tenocytes rats, untreated rats, and uninjured rats at weeks 2 after transplantation.

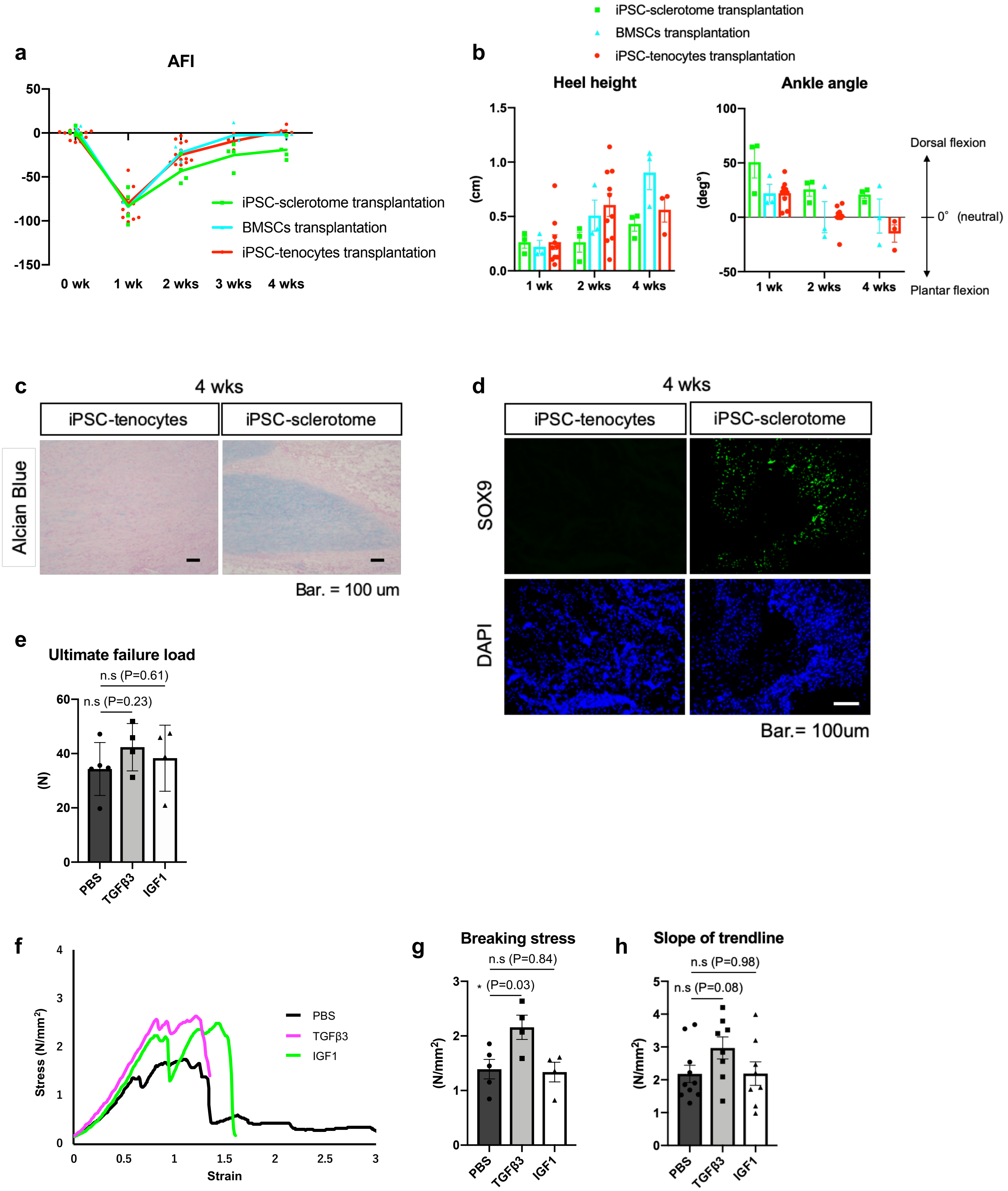

Figure S4. Therapeutic effects of iPSC-derived sclerotome, BMSC, and recombinant TGFβ3, IGF1 proteins

a-b, Kinematic analyses of rats treated with iPSC-tenocytes, iPSC-derived sclerotome or BMSC. AFI was measured at weeks 0 (before surgery), 1, 2, 3, and 4 after transplantation. A value of 0

indicates normal function and a negative value indicates impairment (iPSC-tenocytes: 0, 1, 2 weeks: n = 14: biologically independent samples; 3 and 4 weeks: n = 4: biologically independent samples; iPSC-derived sclerotome: n = 4: biologically independent samples; BMSC: n = 3: biologically independent samples) (**a**). Heel height (cm) and ankle angle (deg°) of walking rats on a treadmill were assessed at weeks 1, 2, and 4 after transplantation. Positive angle indicates the dorsal flexion, on the other hand, negative angle indicates the plantar flexion (**b**). Error bars: mean ± SE (iPSC-tenocytes: 1 and 2 weeks: n = 10: biologically independent samples; 4 weeks: n = 3: biologically independent samples; iPSC-derived sclerotome: n = 3: biologically independent samples; BMSC: n = 3: biologically independent samples). Datasets of iPSC-tenocytes used here for AFI (a), heel height and ankle angle (b) are same as Fig.4c and 4d. **c,** Alcian blue staining of regenerated Achilles tendons harvested from iPSC-tenocytes rats and iPSC-derived sclerotome rats at weeks 4 after transplantation. **d**, Immunohistochemistry of regenerated Achilles tendons harvested from iPSC-tenocytes rats and iPSC-derived sclerotome rats at 4 weeks after transplantation. Tissues were stained with anti-SOX9 (green) antibody and DAPI (blue). **e**, Biomechanical analysis of rats treated with recombinant TGFβ3 and IGF1 proteins. Ultimate failure load (N) of regenerated tendons harvested from rats were assessed at weeks 2 after transplantation. Error bars: mean ± SE (PBS: n = 5: biologically independent samples; TGFβ3 and IGF1: n = 4: biologically independent samples). **f-h**, Representative stress-strain curves (**f**), breaking stress (N/mm^2) (**g**), and slope of a trendline drawn along linear portion of the stress-strain curve (strain < 0.5) (**h**) of repaired tendon in rats treated with recombinant TGFβ3 or IGF1 proteins, or PBS at weeks 2 after transplantation. Error bars: mean ± SE (PBS: n = 5 or 10: biologically independent samples; TGFβ3 and IGF1: n = 4 or 8: biologically independent samples). *$P < 0.05$, two-tailed Welch's t-test. n.s, not significant. Source data are provided as a Source Data file.

Supplementary Table S1. Primer sequences for qPCR analysis

Name	Forward	Reverse
ACTB	CACCATTGGCAATGAGCGGTTC	AGGTCTTTGCGGATGTCCACGT
PAX1	CGTCAGCATCCCGCGCTCAT	ACACGCCGTGCTGGTTGGAG
SCX	CCCAAACAGATCTGCACCTTC	GCGAATCGCTGTCTTTCTGTC
MKX	CGCACAGACACTCTGGAAAA	AGCGGCACTTTGACAGTCTT
TNMD	ATGCCTTGTAACTGGTGGGT	AGGGGCCAGATGCCTCATAG
COL1A1	GGACACAGAGGTTTCAGTGGT	GCACCATCATTTCCACGAGC
COL1A2	GGATGAGGAGACTGGCAACC	TTGCCCTCAGCAACAAGTTC
FMOD	GTGGTGGACGTCGTGAACTT	CCATCAAGCCAAATGCCACG

Supplementary Table S2. Antibodies for immunostaining

	Name	Hosts	Company	Cat. No	Dilution
1st	SCX	Rabbit	abcam	ab58655	1/50
	MKX	Rabbit	Atlas antibodies	HPA006927	1/50
	COL1A1/COLLAGEN I	Rabbit	abcam	ab34710	1/100
	COL1A2	Rabbit	abcam	ab96723	1/100
	Human VIMENTIN	Mouse	abcam	ab230171	1/1000
	COLLAGEN III	Rabbit	abcam	ab7778	1/100
	SOX9	Rabbit	abcam	ab185966	1/200
2nd	Novex Goat anti Mouse IgG(H+L) seondary antibody555		Invitrogen	A21422	1/500
	Novex Goat anti Rabbit IgG(H+L) seondary antibody555		Invitrogen	A21428	1/500
	Novex Goat anti Rabbit IgG(H+L) seondary antibody488		Invitrogen	A11008	1/500
