# Supplementary material for: Grafting of iPS cell-derived tenocytes promotes motor function recovery after Achilles tendon rupture
Source: Nat Commun. 2021 Aug 18;12:5012. doi: 10.1038/s41467-021-25328-6 (PMC8373964; doi:10.1038/s41467-021-25328-6)
Supplement: Supplementary file 3 — Description of Additional Supplementary Files [file 41467_2021_25328_MOESM3_ESM.docx]

Description of Additional Supplementary Files

Title: Supplementary Movie 1

Description: Gait analysis of iPSC-tenocytes rats at two weeks after surgery

Title: Supplementary Movie 2

Description: Stick figure of gait analysis of iPSC-tenocytes rats at two weeks after surgery

Title: Supplementary Movie 3

Description: Gait analysis of untreated rats at two weeks after surgery

Title: Supplementary Movie 4

Description: Stick figure of gait analysis of untreated rats at two weeks after surgery
