# Supplementary material for: Grafting of iPS cell-derived tenocytes promotes motor function recovery after Achilles tendon rupture
Source: Nat Commun. 2021 Aug 18;12:5012. doi: 10.1038/s41467-021-25328-6 (PMC8373964; doi:10.1038/s41467-021-25328-6)
Supplement: Supplementary file 8 — Reporting Summary [file 41467_2021_25328_MOESM8_ESM.pdf]

nature research

Corresponding author(s): Taiki Nakajima
Makoto Iketa

Last updated by author(s): Jul 29, 2021

Reporting Summary

Nature Research wishes to improve the reproducibility of the work that we publish. This form provides structure for consistency and transparency in reporting. For further information on Nature Research policies, see our Editorial Policies and the Editorial Policy Checklist.

Statistics

For all statistical analyses, confirm that the following items are present in the figure legend, table legend, main text, or Methods section.

n/a	Confirmed	
☐	☒	The exact sample size (n) for each experimental group/condition, given as a discrete number and unit of measurement
☐	☒	A statement on whether measurements were taken from distinct samples or whether the same sample was measured repeatedly
☐	☒	The statistical test(s) used AND whether they are one- or two-sided<br>*Only common tests should be described solely by name; describe more complex techniques in the Methods section.*
☒	☐	A description of all covariates tested
☒	☐	A description of any assumptions or corrections, such as tests of normality and adjustment for multiple comparisons
☐	☒	A full description of the statistical parameters including central tendency (e.g. means) or other basic estimates (e.g. regression coefficient) AND variation (e.g. standard deviation) or associated estimates of uncertainty (e.g. confidence intervals)
☐	☒	For null hypothesis testing, the test statistic (e.g. F, t, r) with confidence intervals, effect sizes, degrees of freedom and P value noted<br>*Give P values as exact values whenever suitable.*
☒	☐	For Bayesian analysis, information on the choice of priors and Markov chain Monte Carlo settings
☒	☐	For hierarchical and complex designs, identification of the appropriate level for tests and full reporting of outcomes
☒	☐	Estimates of effect sizes (e.g. Cohen's d, Pearson's r), indicating how they were calculated

Our web collection on statistics for biologists contains articles on many of the points above.

Software and code

Policy information about availability of computer code

Data collection	BZ-H3A (BZ-X700)
Data analysis	StepOne software v2.1, ImageJ v1.52, CellRanger software v3.1.0, Seurat v3.1.1 R packages on R v3.6.1, KineAnalyzer v4.4.1.1910, Living Image Software v4.5, ProteinPilot v5.0, Mascot v2.5, GraphPad Prism8 v8.1.1

For manuscripts utilizing custom algorithms or software that are central to the research but not yet described in published literature, software must be made available to editors and reviewers. We strongly encourage code deposition in a community repository (e.g. GitHub). See the Nature Research guidelines for submitting code & software for further information.

Data

Policy information about availability of data

All manuscripts must include a data availability statement. This statement should provide the following information, where applicable:

- Accession codes, unique identifiers, or web links for publicly available datasets
- A list of figures that have associated raw data
- A description of any restrictions on data availability

The accession number for single-cell RNA sequencing data reported in this study is GSE156753 (https://www.ncbi.nlm.nih.gov/geo/query/acc.cgi?acc=GSE156753). The MS/MS data were deposited in ProteomeXchange Consortium via jPOSTrepo61 with a dataset identifier JPST000939 (https://repository.jpostdb.org/entry/JPST000939).

Field-specific reporting

Please select the one below that is the best fit for your research. If you are not sure, read the appropriate sections before making your selection.

- [x] Life sciences
- [ ] Behavioural & social sciences
- [ ] Ecological, evolutionary & environmental sciences

For a reference copy of the document with all sections, see nature.com/documents/nr-reporting-summary-flat.pdf

Life sciences study design

All studies must disclose on these points even when the disclosure is negative.

Sample size	Required sample sizes were estimated based on established protocols in the research field (Murrell G.A et al., J Orthop Res, 1992; Huang T.F et al., J Sports Med, 2013; Wang T et al., PLoSOne, 2018). The sample sizes were adequate as the differences between experimental groups were reproducible. We ensured that addition of sample sizes does not significantly affect the statistical outcomes. All n values are reported in the figure legends.
Data exclusions	On QC step of the scRNA sequencing, low complexity transcriptomes were filtered out by excluding cell barcodes associated with < 250 genes, < 10,000 reads or > 25% of mitochondrial transcripts. During proteomics data analysis, the expression level indicating < 100 in any one of iPSCs-tenocytes, iPSCs-sclerotome, BMSC were filtered out. Such QC steps were widely applied to cut off unreliable values (e.g Diaz-Cuadros M et al., Nature 2020; Osorio D et al., bioRxiv, 2020).
Replication	To ensure the reproducibility data, we performed all experiments several independent times. Exact number of n for each experiment was written in the figure legends. Each independent experiment contains technical triplicates. We confirmed that these independent datasets did not change the interpretation of results.
Randomization	For all animal experiments, rats were randomly divided into eight treatment groups: iPSC-tenocytes group, iPSC-derived sclerotome group, BMSCs group, untreated group, uninjured group, IGF1 injection group, TGFβ3 injection group, and PBS injection group. To calculate Achilles Functional Index, each footprint collected from rats were randomly selected by a third party who is not listed as an author.
Blinding	For all experiments that count the number of cells (cell density, positive rate, e.g. Figure 1c, Figure 1d), each sample used for the analysis was randomly selected by a third party who is not listed as an author. For animal experiments, rat's footprints used for the analysis were randomly selected by a third party who is not listed as an author.

Reporting for specific materials, systems and methods

We require information from authors about some types of materials, experimental systems and methods used in many studies. Here, indicate whether each material, system or method listed is relevant to your study. If you are not sure if a list item applies to your research, read the appropriate section before selecting a response.

Materials & experimental systems

n/a	Involved in the study	
[ ]	[x]	Antibodies
[ ]	[x]	Eukaryotic cell lines
[x]	[ ]	Palaeontology and archaeology
[ ]	[x]	Animals and other organisms
[x]	[ ]	Human research participants
[x]	[ ]	Clinical data
[x]	[ ]	Dual use research of concern

Methods

n/a	Involved in the study	
[x]	[ ]	ChIP-seq
[x]	[ ]	Flow cytometry
[x]	[ ]	MRI-based neuroimaging

Antibodies

Antibodies used	SCX: Rabbit, abcam, ab58655, Lot.GR212990-6, 1/50; MKX: Rabbit, Atlas antibodies, HPA006927, Lot.A83377, 1/50; COL1A1/COLLAGEN I: Rabbit, abcam, ab34710, Lot.GR255163-3 and GR3241980-1, 1/100; COL1A2: Rabbit, abcam, ab96723, Lot.GR272383-4, 1/100; Human VIMENTIN: Mouse, abcam, ab230171, LN-6, Lot.GR3267195-2, 1/1000; COLLAGEN III: Rabbit, abcam, ab7778, Lot.GR3208254-5, 1/100; SOX9: Rabbit, abcam, ab185966, EPR14335-78, Lot.GR3241181-13, 1/200
Validation	All antibodies were validated by suppliers accordingly. SCX: ab58655 is recommended for detection of mouse, dog, human SCX by ICC (Immunocytochemistry), IHC (Immunohistochemistry), and WB (Wester Blot); MKX: A83377 is recommended for detection of human MKX by ICC and IHC; COL1A1/COLLAGEN I: ab34710 is recommended for detection of human, cow, mouse COL1A1 by IHC and WB; COL1A2: ab96723 is recommended for detection of human COL1A2 by ICC, IHC, and WB; VIMENTIN: ab230171 is recommended for detection of human VIMENTIN by ICC, IHC, and WB; COLLAGEN III: ab7778 is recommended for detection of cow, human COLLAGEN III by IHC and WB.

Eukaryotic cell lines

Policy information about cell lines

Cell line source(s)	Human iPS cell line 1231A3 was established at Center for iPS Cell Research and Application, Kyoto University.
Authentication	Authentication was unnecessary due to the unique morphology and differentiation potential of the iPSCs.
Mycoplasma contamination	The cell line tested negative for mycoplasma contamination.
Commonly misidentified lines (See ICLAC register)	No commonly misidentified cell lines were used in this study.

Animals and other organisms

Policy information about studies involving animals; ARRIVE guidelines recommended for reporting animal research

Laboratory animals	Eight-week-old male F344/Nslc rats
Wild animals	This study does not involve wild animals.
Field-collected samples	This study does not involve samples collected from the field.
Ethics oversight	All animal experiments were approved by the Institutional Animal Committee of Kyoto University.

Note that full information on the approval of the study protocol must also be provided in the manuscript.
